# Unveiling the mysteries of *HvANS*: a study on anthocyanin biosynthesis in qingke (*hordeum vulgare* L. var. *Nudum* hook. f.) seeds

**DOI:** 10.1186/s12870-024-05364-2

**Published:** 2024-07-06

**Authors:** Yan Wang, Youhua Yao, Yongmei Cui, Likun An, Xin Li, Yixiong Bai, Baojun Ding, Xiaohua Yao, Kunlun Wu

**Affiliations:** 1 Qinghai Academy of Agricultural and Forestry Sciences, Qinghai University, Xining, Qinghai China; 2 Qinghai Key Laboratory of Hulless Barley Genetics and Breeding, Xining, Qinghai China; 3 Qinghai Subcenter of National Hulless Barley Improvement, Xining, Qinghai China; 4 Laboratory for Research and Utilization of Qinghai Tibet Plateau Germplasm Resources, Xining, Qinghai China

**Keywords:** Qingke, *HvANS* gene, Anthocyanin, Expression pattern, Functions and mechanisms

## Abstract

**Background:**

Based on our previous research, a full-length cDNA sequence of *HvANS* gene was isolated from purple and white Qingke. The open reading frame (ORF) in the purple variety Nierumuzha was 1320 base pairs (bp), encoding 439 amino acids, while the ORF in the white variety Kunlun 10 was 1197 bp, encoding 398 amino acids. A nonsynonymous mutation was found at the position of 1195 bp (T/C) in the coding sequence (CDS) of the *HvANS* gene. We carried out a series of studies to further clarify the relationship between the *HvANS* gene and anthocyanin synthesis in Qingke.

**Results:**

The conservative structural domain prediction results showed that the encoded protein belonged to the PLN03178 superfamily. Multiple comparisons showed that this protein had the highest homology with *Hordeum vulgare*, at 88.61%. The approximately 2000 bp promoter sequence of the *HvANS* gene was identical in both varieties. The real-time fluorescence PCR (qRT-PCR) results revealed that *HvANS* expression was either absent or very low in the roots, stems, leaves, and awns of Nierumuzha. In contrast, the *HvANS* expression was high in the seed coats and seeds of Nierumuzha. Likewise, in Kunlun 10, *HvANS* expression was either absent or very low, indicating a tissue-specific and variety-specific pattern for *HvANS* expression. The subcellular localization results indicated that HvANS was in the cell membrane. Metabolomic results indicated that the *HvANS* gene is closely related to the synthesis of three anthocyanin substances (Idaein chloride, Kinetin 9-riboside, and Cyanidin O-syringic acid). Yeast single hybridization experiments showed that the *HvANS* promoter interacted with HvANT1, which is the key anthocyanin regulatory protein. In a yeast two-hybrid experiment, we obtained two significantly different proteins (ZWY2020 and POMGNT2-like) and verified the results by qRT-PCR.

**Conclusions:**

These results provide a basis for further studies on the regulatory mechanism of *HvANS* in the synthesis of anthocyanins in Qingke purple grains.

**Supplementary Information:**

The online version contains supplementary material available at 10.1186/s12870-024-05364-2.

## Background

Qingke (*Hordeum vulgare* L. var. *nudum* Hook. f.), also known as naked barley or hulless barley, is a crop of the Gramineae family and is a one- or two-year-old herb, which can be divided into two-row and six-row types [1]. The nutritional value of barley is high, with the nutritional characteristics of “three highs and two lows,” of which the “three highs” refer to high fiber, high protein, and high vitamins and the “two lows” refer to low fat and low sugar; thus, long-term consumption of barley is extremely beneficial to the nutritional structure of the human diet [2]. China is rich in barley varieties. As different pigments are deposited in the pericarp and dextrin layer of barley seeds, resulting in the presentation of different colors, the colors of Qingke seeds are currently mainly classified as blue, black, and purple [3, 4]. Compared to common barley, colored barley is rich in beneficial components such as anthocyanins, phenolic compounds, proteins, and some trace elements, which has led to an increasing emphasis on research into colored barley [5].

Anthocyanins and proanthocyanidins (PAs) are two of the main flavonoids, of which anthocyanins are water-soluble natural pigments found widely in plants and are involved in many plant functions [6]. There are more than 20 known anthocyanins, six of which are more common in plants: geranophyllin, anthocyanin, delphinidin, peonidin, petunidin, and mallowin [7]. Research has shown that anthocyanins not only have various functions, such as antioxidant and anti-tumor properties and cardiovascular disease and Alzheimer’s disease reduction, but can also be used as a coloring agent for a variety of colors in food processing, meeting people’s requirements for healthy, nutritious, and green development [8–11]. Previous studies have also demonstrated the effectiveness of anthocyanins in protecting against ultraviolet light exposure and in reducing skin surface damage caused by radiation. They can be used in the production of cosmetics and related cosmetic products [12]. The anthocyanin biosynthetic pathway, key enzymes, and factors affecting anthocyanin content in fruits have been studied [13–15].

Anthocyanidin synthase (ANS) is a 2-ketoglutarate-dependent dioxygenase, also known as leucoanthocyanidin dioxygenase (LDOX), which is a key enzyme in the anthocyanin synthesis pathway and responsible for the conversion of colorless anthocyanins to colored anthocyanins [16, 17]. The gene encoding ANS has been isolated from many plant varieties, including grapes, mustard, and purple-fleshed sweet potatoes [16, 18, 19]. The expression pattern of the *ANS* gene has been detected in different organs and tissues of various plant varieties [16, 18]. Researchers have revealed ANS expression patterns in grape and purple-fleshed sweet potatoes during fruit development [16, 18]. In begonia, an increased accumulation of anthocyanins and a deeper red color have been found in the petals of plants overexpressing *ANS* compared to the control [20]. At present, the regulation of ANS expression is influenced by a variety of factors, such as the modification of sequence methylation, insertion and deletion of nucleotides in the coding region, transcription factors (TFs), and various environmental factors, and these include both positive and negative regulation [21–23]. We previously obtained a differentially expressed gene, *HvANS*, using transcriptome sequencing of Qingke varieties with different grain colors [1]. However, the expression patterns of *ANS* in different seed color varieties and development stages are still unknown in Qingke. In this study, for the first time, we isolated the promoter sequence of *HvANS* from Qingke. We then determined the expression pattern of *HvANS* in different tissues and developmental stages. Finally, a partial study of its expression regulation mechanism was carried out. The results lay a foundation for further research on the regulatory mechanism of *HvANS* in the anthocyanin synthesis of purple-seeded Qingke.

## Results

### Analysis of HvANS protein physicochemical properties

We isolated the *HvANS* gene from Nierumuzha and Kunlun 10 in our previous study (Yao et al., 2022). The seed coat cDNA of Nierumuzha and Kunlun 10 was used as a template for PCR amplification, and a band of approximately 1300 bp was obtained by 2% gel electrophoresis (Figure S1 A). The recovered product was ligated with the pEasy-Blunt vector (TransGen, China) and transformed into multiple *Escherichia coli* Trans-T1 receptor cells, and three positive clones were selected for sequencing. The sequencing results showed that the CDS of Nierumuzha *HvANS* was 1320 bp in length and that the gene encoded 439 amino acids (Figure S1 B). The CDS length of Kunlun 10 *HvANS* was 1197 bp, and the gene encoded 398 amino acids (Figure S1 B). Kunlun 10 had a non-synonymous mutation (T/C) at position 1195 of the CDS in the *HvANS* gene.

**Fig. 1:**
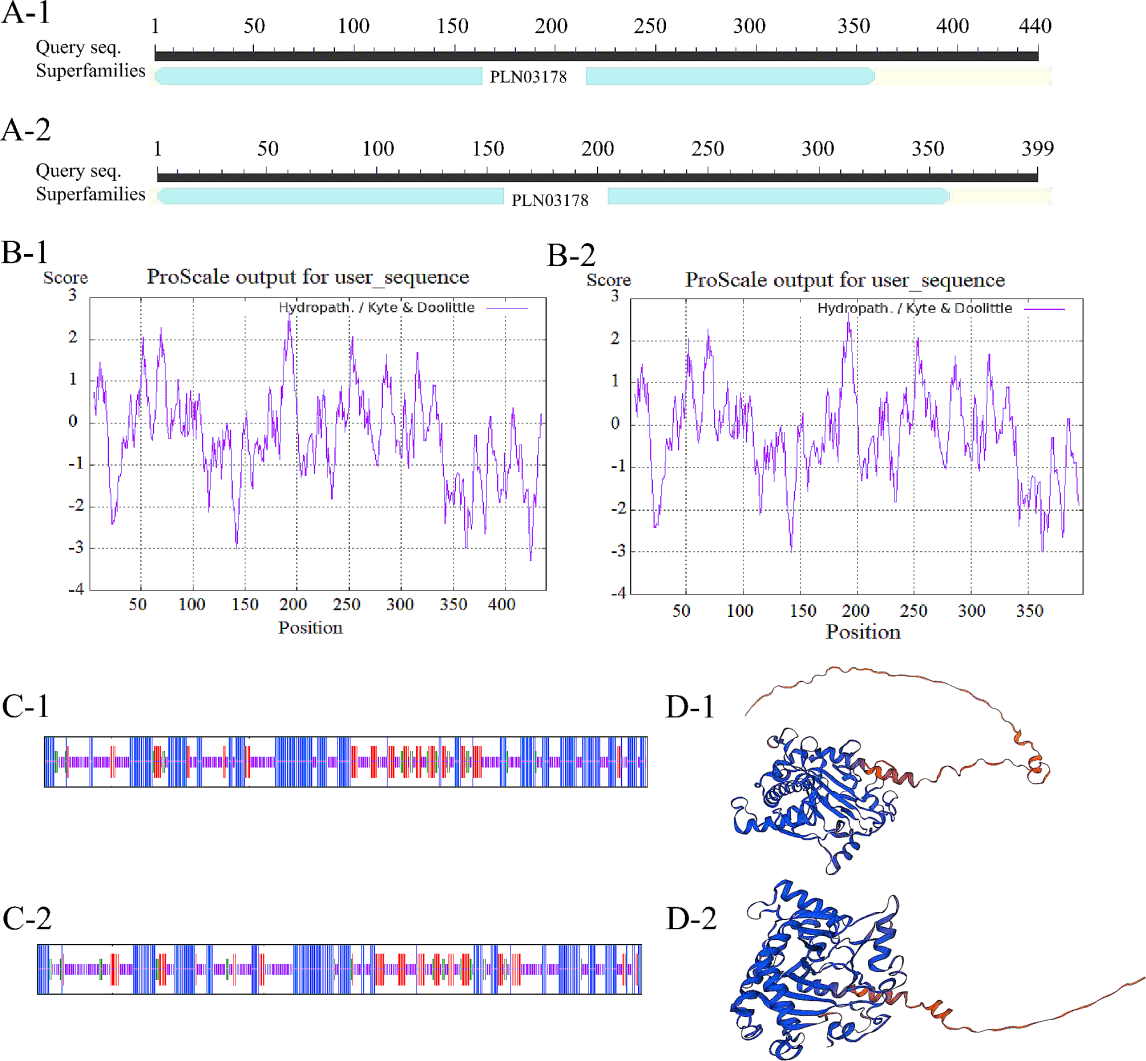
Bioinformatics analysis of the *HvANS* gene CDS. **(A)** Prediction of conserved structural domains of HvANS proteins. **(B)** Prediction of hydrophilicity and hydrophobicity of HvANS proteins. **(C)** Secondary structure prediction of the HvANS protein. **(D)** Tertiary structure prediction of the HvANS protein. 1 is Nierumuzha, 2 is Kunlun 10

The amino acid sequences of the two varieties were predicted using a CD search in NCBI for the conserved structural domains of the gene, and the results showed that the protein encoded by the gene in both varieties belonged to the PLN03178 superfamily (Fig. 1A). The physicochemical properties were predicted using the online tool Protparam, which showed that the Nierumuzha HvANS protein had a molecular formula of C_2114_H_3336_N_588_O_657_S_6_, a molecular weight of 47,693.65 Da, an instability index of 49.58, and a lipolysis index of 90.00. The theoretical isoelectric point was 4.80, with 75 negatively charged residues and 74 positively charged residues. The molecular formula of the Kunlun 10 HvANS protein was C_3320_H_5445_N_1197_O_1358_S_423_; this protein had a molecular weight of 97419.33 Da, an instability index of 50.38, a lipolysis index of 15.29, a theoretical isoelectric point of 4.94, and all 0 negative charge residues.

Prediction of the hydrophilicity of the HvANS protein showed that in Nierumuzha, the protein is a lipophilic unstable protein (Fig. 1B) with no signal peptide or transmembrane structure. In Kunlun 10, the protein was a hydrophilic unstable protein (Fig. 1B).

Prediction of the secondary structure of the ANS protein in Nierumuzha revealed that the secondary structures were, in order, randomly coiled (41.69%), α-helix (40.09%), extended chain (13.90%), and β-turn (4.33%) (Fig. 1C). Tertiary predictions for the HvANS protein showed that the HvANS protein was an oligomeric monomer containing an α-helix with a randomly coiled structure (Fig. 1D). The predicted secondary structure of the ANS protein in Kunlun 10 showed that the secondary structures were, in order, random coiling (31.41%), α-helix (40.7%), extended chain (16.58%), and β-turn (11.31%) (Fig. 1C). Tertiary predictions for the HvANS protein in Kunlun 10 showed that the HvANS protein was an oligomeric monomer containing an α-helix with a randomly coiled structure (Fig. 1D).

### Comparative homology and phylogenetic analysis of ANS proteins

**Fig. 2:**
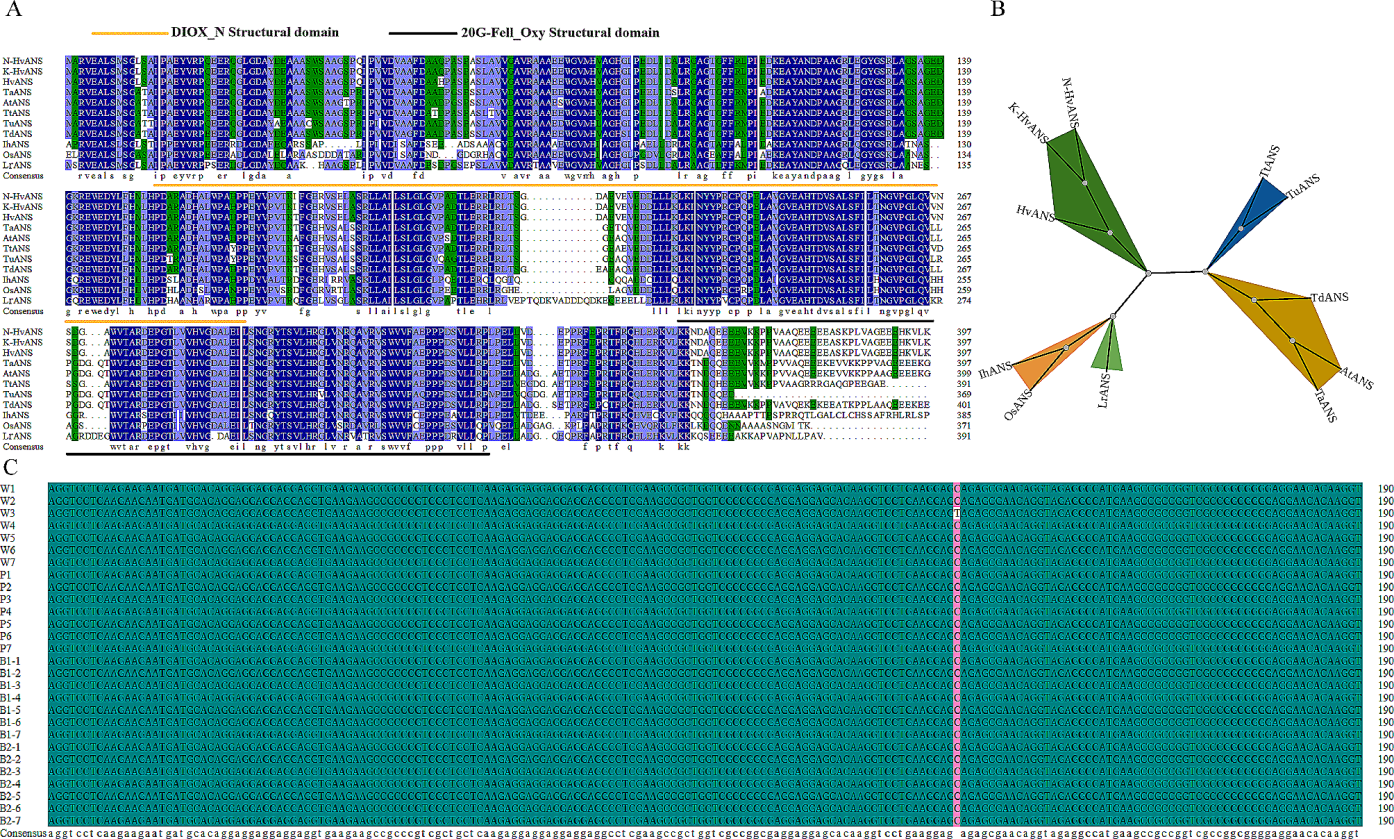
Homology comparison and phylogenetic analysis of ANS proteins. **(A)** Homologous comparison of ANS proteins. **(B)** Phylogenetic analysis of ANS proteins. **(C)** Validation of base mutation loci for 28 Qingke materials. N-HvANS, *Hordeum vulgare* L. var. *nudum* Hook. f. ‘Nierumuzha’; K-HvANS, *Hordeum vulgare* L. var. *nudum* Hook. f. ‘Kunlun 10’; Hv, *Hordeum vulgare* (Ensembl: HORVU.MOREX.r3.5HG0509790); Td, *Triticum dicoccoides* (GeneBank: XP_037444903.1); At, *Aegilops tauschii* (GeneBank: XP_020171118.1); Ih, *Indosasa hispida* (GeneBank: AHC07955.1); Os, *Oryza sativa* Japonica Group (GeneBank: CAA69252.1); Ta, *Triticum aestivum* (GeneBank: AXM42875.1); Tu, *Triticum Urartu* (GeneBank: XP_048537655.1); Tt, *Triticum turgidum* subsp. Durum (GeneBank: VAI52396.1); Lr, *Lolium rigidum* (GeneBank: XP_047057945.1)

The results of the protein multiple sequence comparison showed that the HvANS protein sequence had the highest protein sequence identity with barley ANS (HORVU5Hr1G094280, HORVU.MOREX.r3.5HG0509790) at 99.5%, followed by common wheat (AXM42875.1; 86.32%) and wild tetraploid dicot wheat (XP_037444903.1; 85.68%). The lowest identity was found with *Puccinia* (AHC07955.1) and rice (CAA69252.1), at 57.49% and 56.18% identity, respectively (Fig. 2A). Phylogenetic analyses showed that ANS (N-HvANS and K-HvANS) proteins in Nierumuzha and KL10 were the least genetically distant from ANS proteins in barley (Fig. 2B).

### Validation of SNP mutation loci variation in different seed color resources

To determine whether the color differences between varieties were due to a mutation at position 1195 (T/C) in the *HvANS* gene CDS in Kunlun 10, we tested 28 different colored Qingke materials at the same locus and verified the results, as shown in Fig. 2C. Only the white-3 variety had the same mutation as Kunlun 10, and no mutations were observed in the remaining materials. In addition, no mutations were observed in the remaining six white materials.

### Isolation and sequence analysis of the promoter of *HvANS*

The DNA of barley varieties Nierumuzha and Kunlun 10 was used as the template, and after PCR reactions, a target fragment of about 2000 bp was amplified (Figure S1 C). The sequencing results showed that the *HvANS* promoter sequences were identical in both varieties. After online prediction of the promoter transcription start site, there were three possible core regions in the promoter region of the Qingke *HvANS* gene, located at 39–89, 440–490, and 1904–1954 bp, with scores of 0.88, 0.98, and 0.95, respectively, and the possible transcription start sites were A, T, and A, respectively. This led to the assumption that the sequence at 440–490 bp was the true core promoter region of the gene, with the transcription start site at T at 480 bp (Table 1). Online prediction of promoter biological functions showed that the promoter region of the *HvANS* gene contained a large number of core promoter elements, such as the CAAT-box and TATA-box, in addition to a variety of cis-elements, such as the abscisic acid-related element ABRE, the anaerobic induction-related element ARE, the low-temperature response element LTR, the light response-related elements AE-box and G-box, and the MYB binding site (Table S1).

**Table 1:** Prediction of promoter transcription start sites

Start	End	Score	Promoter Sequence
39	89	0.88	TCAGTGACAATATAAATATAGTTGAGTTAGTGTGTCGGTGACCGAAAGTT
440	490	0.98	CCCTCCCTCCTATATATACTAGAGGAAAGGGAGGGCAGCCTTCCCTCTCT
1904	1954	0.95	CACCAAACAATCTATAAGTAACCCTAAACCCATTCCATCCATGAACTCCA

### Expression profiles of *HvANS* in different tissues of two different varieties

**Fig. 3:**
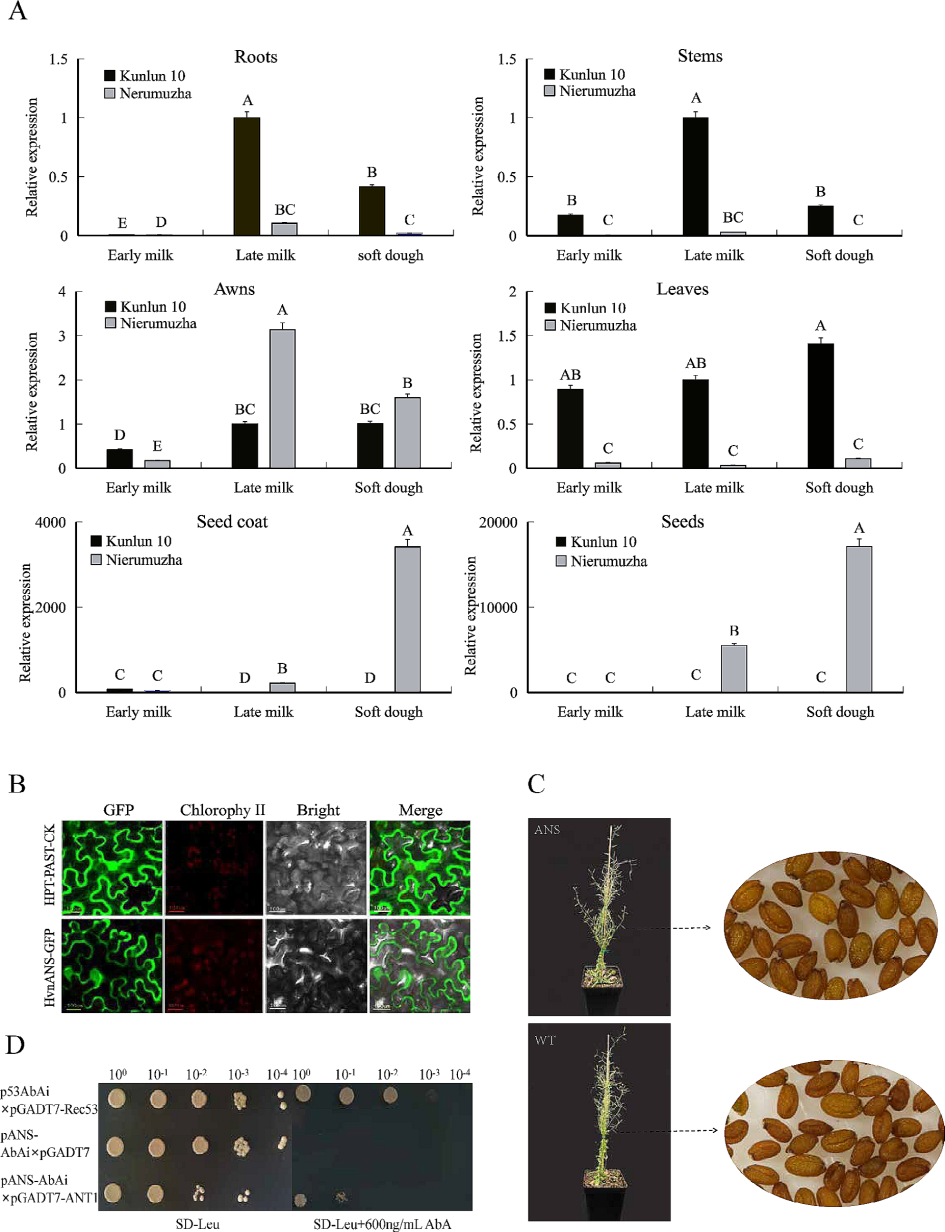
Functional studies of the *HvANS* gene. **(A)** Spatial and temporal expression patterns of *HvANS* in grain color formation of barley varieties with different grain colors. **(B)** Subcellular localization of the HvANS protein. **(C)** Positive transgenic *Arabidopsis thaliana* seedlings and seeds. (**D**)Y1H assay showing the interaction between HvANT1 and the *HvANS* promoter. p53AbAi x pGADT7-Rec53 was used as a positive control, pANS-AbAi x pGADT7 was used as a negative control, and pANS-AbAi x рGADT7-ANT1 was used as an experimental group. Capital letters in graph A represent highly significant differences. GFP, green fluorescence; Chlorophy II, Chloroplast; Bright, bright field; Merge, Superimposed. A, B, C, D. statistically significant (*p* < 0.01). SD-Leu, yeast-deficient medium (without leucine); AbA, gold tamoxifen

We have previously reported that the total anthocyanin content of Nierumuzha was significantly higher than that of Kunlun 10, and this result is important for our subsequent experimental work (Yao et al., 2022). We used qRT-PCR to detect the relative expression of this gene in different tissues (root, stem, leaf, awn, seed, and seed coat) of Nierumuzha and Kunlun 10 during the early lactation, late lactation, and soft dough stages of seed color formation. The results showed that the expression of *HvANS* in Kunlun 10 was low or almost absent in all tissues, while the expression of *HvANS* in Nierumuzha was tissue specific, with extremely low expression in the roots, stems, leaves, and awns, but very high expression in seed coats and seeds. The expression of *HvANS* was significantly higher in the seed coats at the late milk and soft dough stages (*p* < 0.01), reaching the maximum at the soft dough stage, about 15 times higher than that at late lactation and 66 times higher than that at early lactation. *HvANS* expression was also significantly higher in the seeds of Nierumuzha than in the seeds of Kunlun 10 (*p* < 0.01), consistent with its expression pattern in the seed coat, reaching the maximum at the soft dough stage, about five times higher than at the late milk stage (about 900-fold; Fig. 3A). In conclusion, these results indicate that the color presented by the tissues was related to anthocyanin accumulation, and the expression level of HvANS showed significant variation between different tissues of different varieties. *HvANS* gene expression is hypothesized to gradually increase with anthocyanin synthesis in seed coats and seeds, suggesting that this gene may enhance anthocyanin accumulation.

### Subcellular localization of HvANS

As shown in Fig. 3B, the green fluorescent signal of pANS-GFP was mainly present in the cell membrane of tobacco. In contrast, the green fluorescent signal of the control was found throughout the cell, indicating that HvANS is a protein located in both cell membranes.

### HvANS overexpression leads to anthocyanin accumulation in *Arabidopsis* seeds

Transgenic *Arabidopsis thaliana* T_0_ generation lines with the *HvANS* gene were obtained after *Agrobacterium* infestation, healing screening, differentiation, and rooting, as well as after screening and validation of transgenic plants. DNA from the leaves of all transgenic *Arabidopsis* plants was extracted using the CTAB method, and PCR was performed to identify positive seedlings. We eventually obtained positive transgenic *Arabidopsis* plants overexpressing all genes. Successive screening and amplification resulted in T_3_ generation transgenic *Arabidopsis* (Fig. 3C). As observed in the *Arabidopsis* seedlings, there were no phenotypic differences between the wild-type and overexpressing plants.

Since overexpressing *Arabidopsis* did not have significant color differences, we subjected the T_2_ generation seeds of overexpressing *Arabidopsis* to metabolomic assays to determine whether anthocyanin substances changed in the seeds of overexpressing plants. A total of 11 anthocyanin substances were detected (Table 2). Three of the anthocyanin substances (Idaein chloride, Kinetin 9-riboside and Cyanidin O-syringic acid) were significantly elevated (*p* < 0.05) in the seeds of overexpressing plants. This suggests that the *HvANS* gene is closely related to anthocyanin synthesis.

**Table 2:** 11 anthocyanins from overexpressed plants

Name	WT	ANS
Peonidin O-hexoside	83373.33 ± 9441.96	72760.00 ± 12727.89
Delphinidin 3-O-Rutinoside	121700000.00 ± 2843706.50	1296666666.70 ± 5272781.26
Procyanidin A3	335466.67 ± 63634.86	252000.00 ± 52327.49
Petunidin 3-O-glucoside	431466.67 ± 59462.61	435266.67 ± 86571.45
Cyanidin chloride	10031.33 ± 1895.31	7426.67 ± 2182.42
Cyanidin 3-O-glucoside	414100.00 ± 33534.86	506433.33 ± 58666.65
Petunidin-3-O-glucoside	13010.00 ± 1578.44	11129.67 ± 1883.58
Idaein chloride	262933.33 ± 5028.14	329266.67 ± 20172.97*
Luteolin O-hexosyl-O-hexosyl-O-hexoside	54156.67 ± 9716.53	62940.00 ± 1448.59
Cyanidin O-syringic acid	250966.67 ± 22046.67	299200.00 ± 10007.00*
Kinetin 9-riboside	36230.00 ± 5976.97	63350.00 ± 7587.48*

* *p* < 0.05

### HvANT1 binds to the *HvANS* promoter

To determine the reasons for the differences in *HvANS* expression in the two Qingke varieties, a 1997 bp *HvANS* promoter sequence was isolated from Qingke Nierumuzha. The Y1H experiment was then carried out in which the p53-AbAi receptor single-transformed pGADT7-Rec53 plasmid was grown as a positive control on SD-Leu + AbA medium at the lowest inhibitory concentration and without the addition of inhibitors. The pANS-AbAi receptor state single transfer of pGADT7 served as a negative control and did not grow at all on SD-Leu + AbA medium at the lowest inhibitory concentration. In the experimental group, pANT1-GADT7 and pANS-AbAi vectors were co-transformed into yeast. pANT1-GADT7 and pANS-AbAi could be grown on a selective SD-Leu + AbA medium, indicating that the CDS region of *HvnANT1* interacts with the promoter region of *HvANS* (Fig. 3D).

### HvANS interacts with two proteins

**Fig. 4:**
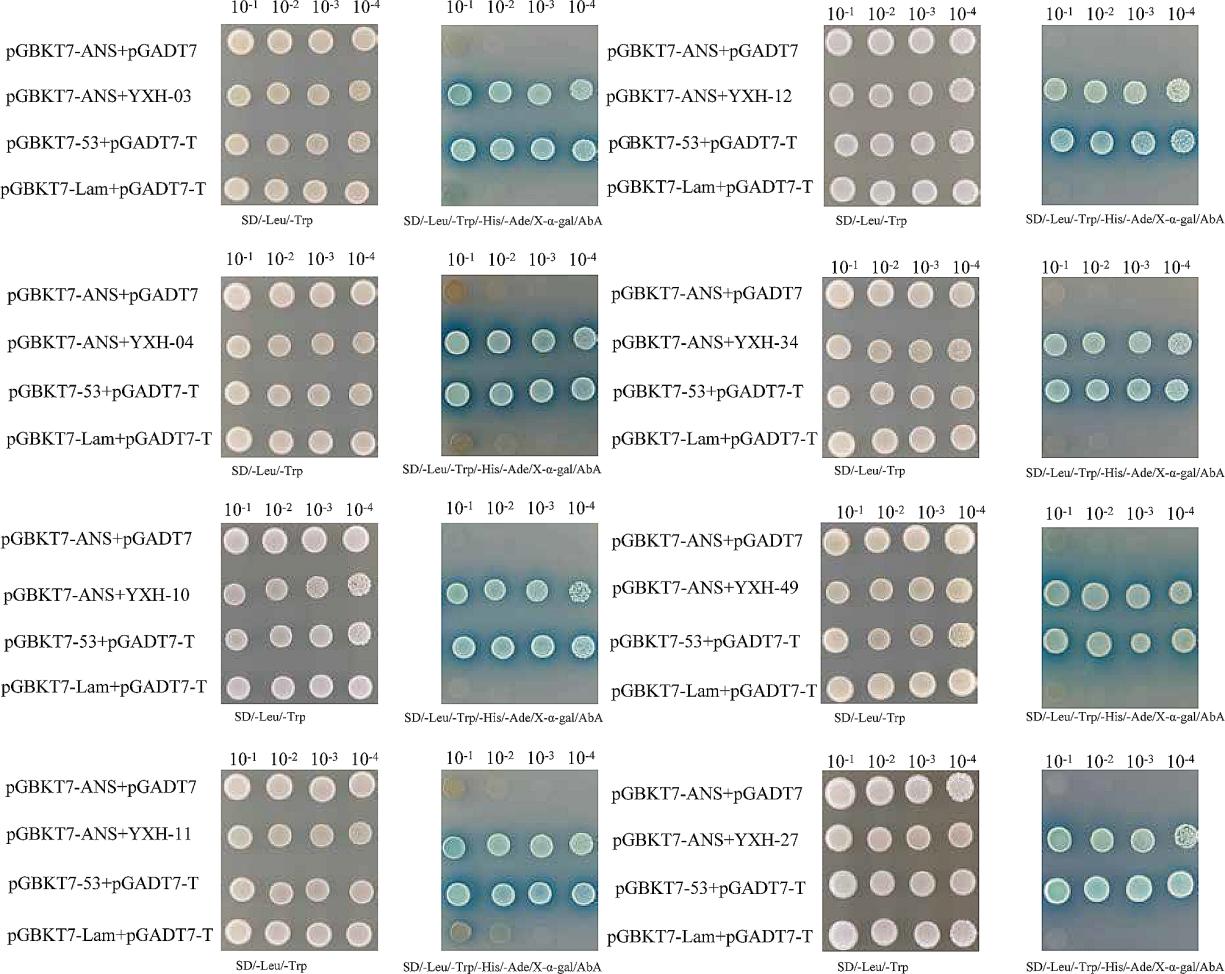
Interactions of the HvANS protein with eight proteins revealed by yeast two-hybrid experiments

**Fig. 5:**
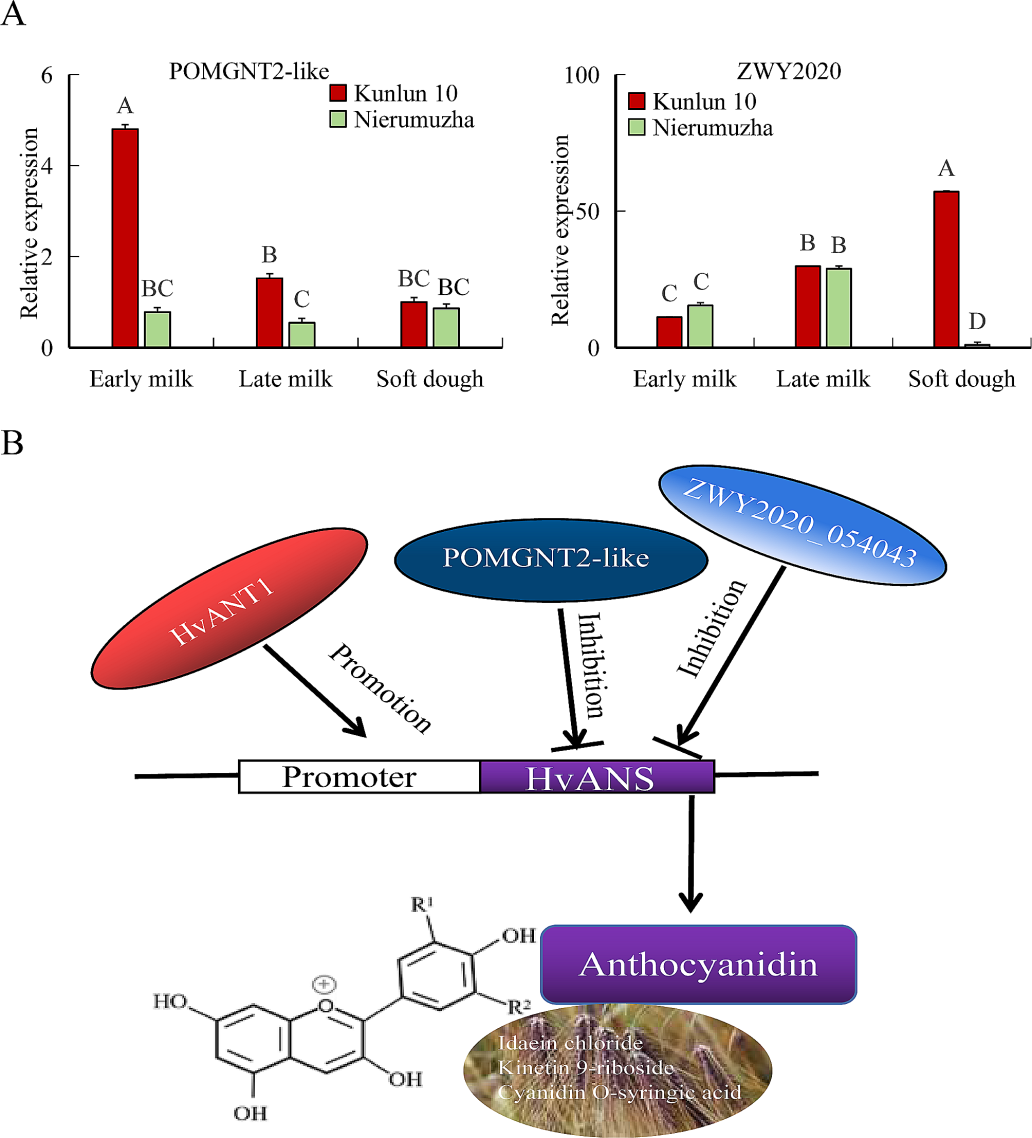
**(A)** Expression of two differentially expressed proteins in two varieties. **(B)** DNA pattern of ANS-interacting proteins in purple Qingke. A, B, C, D. statistically significant (*p* < 0.01)

We constructed a yeast hybrid library for Nierumuzha. The quality of the nuclear yeast library was tested. The results of the library plasmid quality check are shown in Table 3. The full-length CDS of *HvANS* was cloned into the pGBKT7 vector to generate a decoy plasmid, which was used to screen for protein interactions by pairing the decoy protein with the library. Finally, 45 blue colonies representing potentially positive clones were obtained on SD/-Leu/-Trp/-His/-Ade/X-α-Gal/AbA plates (Figure S2). The resulting PCR products were sequenced, and sequences were aligned against the barley database using the Basic Local Alignment Search Tool (BLAST) on the National Center for Biotechnology Information website (http://www.ncbi.nlm.nih.gov). Finally, eight interacting protein candidates of interest were identified: HORVU5Hr1G124350, HORVU4Hr1G074250 (HORVU.MOREX.r3.4HG0402730), HORVU0Hr1G006810 (HORVU.MOREX.r3.3HG0282680), HORVU3Hr1G019590 ( HORVU.MOREX.r3.3HG0236690), HORVU3Hr1G098160 (HORVU.MOREX.r3.3HG0313780 ), HORVU4Hr1G003060 (HORVU.MOREX.r3.4HG0333840), HORVU2Hr1G032690 (HORVU.MOREX.r3.2HG0126880), and HORVU5Hr1G053930 (HORVU.MOREX.r3.7HG0726260) (Table 4). Further, Y2H one-to-one validation results confirmed the interaction between these eight proteins and HvANS (Fig. 4). Finally, we identified two significantly different proteins, the putative protein ZWY2020 and the protein o-linked-mannose beta-1,4-N-acetylglucosaminyltransferase 2-like (POMGNT2-like) protein, by combining transcriptome data with qRT-PCR. The experimental results showed that the expression of *ZWY2020* gradually decreased in Nierumuzha and increased in Kunlun 10 as plant fertility increased, and its expression was significantly different between the two varieties. The expression level of *POMGNT2-like* in Kunlun 10 decreased with the progression of the reproductive period, while it remained consistently low in Nierumuzha. Based on these results, it is hypothesized that the ZWY2020 protein is more likely to be involved in the regulation of anthocyanins (Fig. 5A).

**Table 3:** Library plasmid quality control results

Name	Capacity (cfu)	Reconstitution rate (%)	Average library length (bp)	Library plasmid concentration (ng/uL)	Total library quality (ug)
Primary library	1.28 × 107	100	> 1000	277	277
Nuclear system sub-library	1.12 × 107	100	> 1000	828	828

**Table 4:** Eight interacting proteins

gene number	gene name	gene ID (morex V2)	gene ID (morex V3)	genebank
1	predicted protein [Hordeum vulgare subsp. vulgare]	HORVU5Hr1G124350		BAJ99788.1
2	BEL1-like homeodomain protein 6 [Hordeum vulgare]	HORVU4Hr1G074250	HORVU.MOREX.r3.4HG0402730	KAE8782045.1
3	transcription repressor MYB5-like [Hordeum vulgare]	HORVU0Hr1G006810	HORVU.MOREX.r3.3HG0282680	KAE8819708.1
4	R2R3-MYB protein [Hordeum vulgare]	HORVU3Hr1G019590	HORVU.MOREX.r3.3HG0236690	KAE8802623.1
5	predicted protein [Hordeum vulgare subsp. vulgare]	HORVU3Hr1G098160	HORVU.MOREX.r3.3HG0313780	BAJ97521.1
6	hypothetical protein ZWY2020 [Hordeum vulgare]	HORVU4Hr1G003060	HORVU.MOREX.r3.4HG0333840	KAI4998701.1
7	Ninja-family protein 3 [Hordeum vulgare]	HORVU2Hr1G032690	HORVU.MOREX.r3.2HG0126880	KAE8784954.1
8	protein O-linked-mannose beta-1,4-N-acetylglucosaminyltransferase 2-like [Hordeum vulgare]	HORVU5Hr1G053930	HORVU.MOREX.r3.7HG0726260	KAE8814963.1

## Discussion

ANS is crucial to plant anthocyanin biosynthesis. In this study, the HvANS protein was classified under the PLN03178 superfamily. The HvANS protein sequence shared the highest identity at 88.61% with the barley ANS protein sequence, indicating genetic evolutionary consistency. Our verification of the mutation loci in 28 other different colored Qingke materials showed that the color differences between the purple and white varieties were not due to base mutations. The qRT-PCR results showed that the expression of *HvANS* differed significantly between the two varieties. The expression of HvANS in Kunlun 10 was minimal or almost absent. However, Nierumuzha showed an increasing trend in both the seed coats and seeds. This expression is specific to certain tissues and varieties, with higher levels found in dark/purple, followed by light, and then white/no color. This is consistent with the findings reported thus far. For example, the tissue expression analysis of *IbANS* in sweet potato by Liu et al. showed that *IbANS* was expressed in all tissues of sweet potato, but the highest expression was in the tuber and pericarp [24]. Zhang et al. performed qRT-PCR expression analysis and showed that transcript expression was significantly higher in highly chromogenic potato *StANS* than in the yellow genotype [25].

The localization of a protein in a cell is often related to its function. Therefore, it is important to study the subcellular localization of proteins to explore their functions. At the subcellular level, we found that HvANS was mainly localized to the cell membrane. Currently reported ANSs are mainly located in the cytoplasm and nucleus, such as grapevine ANS [18], *Medicago truncatula* ANS [26], and *Actinidia arguta* AaLDOX [27]. Research has also shown the accumulation of flavonoids in the nucleus, whose role is to protect DNA from UV and oxidative damage, among other functions [28]. However, the accumulation of flavonoid’s role in the cell membrane needs to be further explored.

Using transgenic technology, we successfully developed overexpressing *Arabidopsis* plants. However, these plants did not manifest the anticipated phenotypes. We performed metabolomic assays on the overexpressing *Arabidopsis* plants due to the absence of an apparent color distinction in phenotype between the overexpressing plants and the controls. The metabolomics assay results demonstrated a significant increase in the three anthocyanin substances in the overexpressing plants compared to the non-overexpressing plants. Consequently, we concluded that the *HvANS* gene has a close association with anthocyanin biosynthesis.

To address these findings, we conducted further studies on the promoter and coding regions of the *HvANS* gene. Online software predictions indicate that the sequence at 440–490 bp is likely to be the true core promoter region of the *HvANS* gene, which contains a large number of core promoter elements, such as the CAAT-box and TATA-box, as well as multiple cis-elements, including the MYB binding site. In plants, TFs, such as MYB, bHLH, and WD40, upregulate the expression of structural genes in the anthocyanin biosynthesis pathway [29, 30]. Several studies illustrate the regulatory role of TFs in anthocyanin biosynthesis. For example, Liu et al. upregulated the expression of key structural genes for anthocyanin synthesis, including *F3H*, *DFR*, and *ANS*, by overexpressing the *LcTT8* transcription factor [31]. Wang et al. significantly activated the expression of anthocyanin biosynthesis genes, including *ANS*, by overexpressing *PdMYB118*, resulting in a red phenotype in the leaves of transgenic plants [32]. Zhou et al. found that *Ant1* acted as a master regulator in the activation of anthocyanin biosynthesis and that *CHS*, *F3H*, *DFR*, and *ANS* genes were significantly activated when *ANT1* and *ANT2* were overexpressed [33]. We verified the interaction between the promoter region of *HvANS* and the HvnANT1 protein. However, further studies are needed to determine whether the difference in color between the two varieties is due to the interaction of only one gene, *ANT1*, with the promoter of *HvANS*. In parallel, we constructed a yeast library and performed a Y2H screen of the yeast library, resulting in a total of eight proteins of interest. A comparison of transcriptomic data revealed a significant relationship between the expression of a putative gene *ZWY2020* (Gene ID: HORVU4Hr1G003060, HORVU.MOREX.r3.4HG0333840) and a *POMGNT2-like* gene (Gene ID: HORVU5Hr1G053930, HORVU.MOREX.r3.7HG0726260), which we also confirmed using qRT-PCR. Putative protein ZWY2020 has not been reported thus far, and studies on the POMGNT2 protein have mainly focused on animal pathology studies [34, 35]. The functions of these two proteins in Qingke require further study. However, based on the qRT-PCR results, it seems that the ZWY2020 protein should be of greater interest. Based on these results, it is reasonable to speculate that HvANT1 interacts with the *HvANS* promoter and has the same expression pattern [36] and that *HvANS* is involved in the regulation of anthocyanin biosynthesis (Idaein chloride, Kinetin 9-riboside, and Cyanidin O-syringic acid) in Qingke (Fig. 5B). In addition, the Y2H results showed significant differences between ZWY2020 and POMGNT2-like proteins in the two varieties, and it is assumed that these two proteins may be involved in the regulation of anthocyanin biosynthesis and that both are negatively regulated. These findings provide good data to support our current experimental results, and we will subsequently further validate their specific regulatory mechanisms with the ANS.

DNA methylation occurring at the carbon-5 position of cytosine (C) is essential for epigenetics in the regulation of gene expression in animals and plants [23]. Lin et al. found that metabolite biosynthesis is regulated by gene expression, which is altered by DNA methylation in the promoter region [37]. Deng et al. tested the ANS of two lotus varieties, the red variety Wild Red Lotus and the white variety White Dove Lotus, for methylation and found that the white variety had higher methylation levels than the red variety. They hypothesized that the difference in methylation levels led to a difference in flower color between the red and white varieties [23]. This also provides new directions for future research.

In conclusion, *HvANS* expression is mainly in the seed coats and seeds of Nierumuzha. *HvANS* expression showed varietal and tissue specificity in different varieties and organs, respectively. At the subcellular level, the HvANS protein is localized to the cell membrane. The metabolomic results strongly associated the HvANS gene with the synthesis of the three anthocyanin substances. Furthermore, our findings indicate that the promoter region of *HvANS* interacts with the MYB family of HvANT1 proteins. In the Y2H results, two proteins interacted with the HvANS protein. The experimental results highlight the intricate regulatory network governing anthocyanin synthesis. Our current experimental results contribute to our understanding of the regulatory mechanism of anthocyanin synthesis. Furthermore, we will delve deeper into the exploration and excavation of this regulatory mechanism in future research.

## Conclusions

Based on our previous research findings, with a non-synonymous mutation (T/C) at position 1195 of the CDS in the *HvANS* gene in the white variety Kunlun 10. We carried out bioinformatics analysis on *HvANS*. The 2046 bp promoter sequence of *HvANS* was isolated, and the sequence at 440–490 bp was likely the true core promoter region of the gene. *HvANS* was most abundantly expressed in the seed coats and seeds of Nierumuzha. HvANS proteins were mainly located in the cell membrane. *HvANS* was associated with the synthesis of three anthocyanin substances. The promoter region of *HvANS* interacted with the HvANT1 protein, and we also obtained two significantly different proteins that interacted with the HvANS protein in the Y2H assay.

## Methods

### Plant materials

The purple Qingke variety Nierumuzha and the white Qingke variety Kunlun 10, both from the Academy of Agricultural and Forestry Sciences of Qinghai University (Xining, Qinghai, China), were planted at the barley experimental base of the Academy of Agricultural and Forestry Sciences of Qinghai University in April 2020. Seeds of Nierumuzha and Kunlun 10 were collected at early milk, late milk, and soft dough stages according to the Zadoks growth scale for gene expression analysis [38]. Three biological replicates of each sample were collected, and the roots, stems, leaves, awns, seeds, and seed coats were immediately frozen in liquid nitrogen after sampling and stored at -80 °C.

### Extraction of RNA and DNA

The seed coats of Nierumuzha and Kunlun 10 were quickly ground to powder in liquid nitrogen, and total DNA and RNA were extracted using a Plant Genomic DNA/RNA Extraction Kit (TIANGEN DP320, Beijing, China). The concentrations and purity of DNA and RNA were determined using a NanoPhotometer (Implen Beijing International Trading Co., Beijing, China). The DNA integrity and RNA integrity of the samples were determined by electrophoresis with the aid of 1.2% agarose gel. Reverse transcription of total RNA was performed using a cDNA Synthesis Kit (PrimeScript 1st Strand cDNA Synthesis Kit) (TaKaRa, Ohtsu, Japan), following the manufacturer’s instructions, and stored at -80 °C.

### Base mutation site validation

To identify whether the mutant locus of the *HvANS* gene is the key locus causing the difference between purple and white seeds, we selected 28 Qingke varieties with different grain colors for validation. Validation primers HvANS-JF/JR (Table 5) were designed using Primer 5.0. cDNA from 28 Qingke materials of different colors (seven each of white, purple, blue, and black-grained barley, Table 6) was used as the template for PCR amplification. The subsequent steps were consistent with those of *HvANS* gene cloning.

**Table 5:** The primers used in this study

Primer name	Primer sequence(5ri)	Purpose
*HvANS*-JF	GAAGCTCAAGATCAACTACTA	Mutation site validation
*HvANS*-JR	TACACTTATGTATTATGAG	Mutation site validation
Promoter-F	CGTTATTGACCGTAGA	Promoter amplification
Promoter-R	CCGGTGGTATGCTCTG	Promoter amplification
*HvANS*-SF	GGGGTTCTTCCGCTTGC	Primers for Real-time PCR
*HvANS*-SR	ACTCCCTCTTCCCGTCC	Primers for Real-time PCR
18SrRNA-F	CGGCTACCACATCCAAGGAA	Control primer
18SrRNA-R	GCTGGAATTACCGCGGCT	Control primer
*HvANS*-GF	GGGGACAAGTTTGTACAAAAAAGCAGGCTTCATGGCGCGGGTGGAGGCACTG	Subcellular localization
*HvANS*-GR	GGGGACCACTTTGTACAAGAAAGCTGGGTCTTAATTAACTTCCACCGGCGC	Subcellular localization
*HvANS*-OF	cagtCACCTGCaaaacaacatggcgcgggtggaggcac	Construction of the *HvANS* overexpression vector
*HvANS*-OR	cagtCACCTGCaaaatacattaattaacttccaccggcgcctctc	Construction of the *HvANS* overexpression vector

**Table 6:** 28 different colours of barley material

Serial number	Name	Serial number	Name
White-1	‘Kunlun 14’	Blue-1	‘Ai Ganqi’
White-2	‘ZYM1906’	Blue-2	‘Ganqing 4’
White-3	‘Baiyu’	Blue-3	‘Blue Qingke’
White-4	‘ZDM9837’	Blue-4	‘Zu Zhuochun’
White-5	‘Rice barley’	Blue-5	‘Qianning’
White-6	‘White 91-97-3’	Blue-6	‘Bianba’
White-7	‘Tangmai Qingke’	Blue-7	‘Qushui Qingke’
Purple-1	‘ZYM1849’	Black-1	‘ZYM1897’
Purple-2	‘Chengduo’	Black-2	‘Shuo Banduo’
Purple − 3	‘ZYM1900’	Black-3	‘ZDM6926’
Purple-4	‘Zikezhanima’	Black-4	‘ZDM7021’
Purple-5	‘Murray Qingke’	Black-5	‘ZYM1749’
Purple-6	‘Purple Kangqing’	Black-6	‘ZDM7179’
Purple-7	‘Da zhangzi’	Black-7	‘ZYM1900’

### Isolation of the *HvANS* promoter

We retrieved the ANS promoter sequence, the reference sequence, from the barley genome using Gramene (https://ensembl.gramene.org/index.html). The promoter primer Promoter-F/R (Table 5) for this gene was designed using Primer 5.0, and PCR amplification was carried out using seed coat DNA from Nierumuzha and Kunlun 10 as templates. The obtained target bands were recovered by gel cutting using a DNA Gel Extraction Kit (TIANGEN BIOTECH CO., LTD, Beijing, China), and the concentration and purity of the gel-recovered products were determined using a NanoPhotometer (Implen Beijing International Trading Co., Beijing, China). The recovered products were ligated with the pEasy-Blunt vector (Alltech Biologicals) and transformed into multiple *Escherichia coli* Trans-T1 receptor cells. Finally, the three positive clones obtained from screening were subjected to PCR using M13 as the amplification primer and sent to Qingke Biotechnology Ltd. (Xian, China) for sequencing.

### Bioinformatics analysis of the *HvANS* gene in Qingke

The conserved structural domains of HvANS were analyzed using NCBI. The physicochemical properties and hydrophilicity/hydrophobicity of the proteins were predicted using Expasy Protparma (https://web.expasy.org/protparam/) and Protscale (https://web.expasy.org/protscale/), respectively. The signal peptides and transmembrane structures of the proteins were predicted using SignalP5.0 (https://services.healthtech.dtu.dk/services/SignalP-5.0/) and TMHMM-2.0 (https://services.healthtech.dtu.dk/services/TMHMM-2.0/), respectively. The secondary and tertiary structures were predicted for Qingke HvANS using the online software NPS (https://npsa-prabi.ibcp.fr/cgi-bin/secpred_sopma.plHvn) and SWISS-MODEL software (https://swissmodel.expasy.org/), respectively. The BLASTP tool in NCBI was used to compare amino acid sequences from grasses that are highly homologous to the HvANS protein. A biological phylogenetic tree was constructed using Mega 7.0 software. Cis-acting element varieties and the distributions and biological functions of promoter sequences were predicted using online Plant CARE software, and transcription start sites (http://bioinformatics.psb.ugent.be/webtools/plantcare/html/) and possible core promoter regions were predicted using online software BDGP (https://www.fruitfly.org/seq_tools/promoter.html).

### Expression pattern of the *HvANS* gene in qingke

The quantitative primers HvANS-SF/SR were designed using Primer 5.0, based on the *HvANS* gene sequences obtained by amplification (Table 5). The cDNAs of roots, stems, leaves, awns, seeds, and seed coats of Nierumuzha and Kunlun 10 at early lactation, late lactation, and soft dough stages were used as templates, and 18 S rRNA was selected as the internal reference gene. TaKaRa TB GreenpremixExTaq II fluorescent dye (TaKaRa, Ohtsu, Japan) was used for RT-qPCR using the LightCycler480 System (Roche, Switzerland). The RT-qPCR reaction system was performed following Yao et al. [39]. Expression data were collated and analyzed using Microsoft Excel 2010 and SPSS 22.0 statistical software, respectively, and presented as the mean ± standard deviation (SD). The relative gene expression in barley varieties at different times was calculated using the 2^−ΔΔCt^ formula [40]. All experiments included three biological replicates, and the experimental data were analyzed by analysis of variance (ANOVA) using SPSS 22.0.

### Subcellular localization of *HvANS*

Gateway technology was used to construct the expression vector. Primers with specific sites were designed for *HvANS* (Table 5), and cDNA from Nierumuzha seed coats was used as a template for introductory vector construction. The recovered products were subjected to BP reactions (Table 7) and digested overnight at 25 °C. Several positive clones were selected for bacteriophage PCR validation and sent to Qingke Biotechnology Ltd. (Xian, China) for sequencing. After extraction and identification of the successful plasmids, homologous recombinant LR reactions (Table 8) were performed and overnight at 25 °C. Finally, the colonies were identified by PCR, and the positive clones were sent to Qingke Biotechnology Ltd. (Xian, China) for sequencing. Plasmids with the correct sequencing results were extracted for subsequent experiments.

**Table 7:** BP reaction system

BP reaction system	10 µL
Pdonr221	1 µL
BP	1 µL
1×TE	3 µL
Target gene fragment	5 µL

**Table 8:** LR reaction system

LR reaction system	10 µL
HPT-PSAT-CK	1 µL
LR	1 µL
1×TE	7 µL
Target gene fragment	1 µL

The recombinant plasmid pANS-GFP was transformed into *Agrobacterium tumefaciens* EH105, followed by an expanded culture (including an empty vector) for 2 days. The bacteriophage was collected when the OD600 reached 0.5, and then it was resuspended with the prepared injection solution. The bacteriophage solution was injected into tobacco using a 1-mL syringe and labelling. Microscopic examination was performed using a fluorescent confocal microscope (Nikon A1R, Tokyo, Japan) after 2 days under dark conditions.

### Construction and transformation of the *HvANS* overexpression vector

To create an overexpression vector, the full-length coding sequence of HvANS was inserted into the BsaI/Eco31I site of the pBWA(V)HS-ccdB-Gus vector (Biorun Biotechnology Co., Wuhan, China) using the Golden Gate method, generating the recombinant vector pBWA(V)HS-HvANS-Gus. Primer sequences used for construction are listed in Table 5. Recombinant constructs were selected based on kanamycin resistance and confirmed by DNA sequencing. The overexpression vector plasmid pBWA(V)HS-HvANS-Gus was transferred by electrotransformation into *Agrobacterium tumefaciens* GV3101 (Biorun Biotechnology Co., Wuhan, China), which was then used to inoculate the model plant ecotype *Arabidopsis thaliana* (ecotype Columbia). After screening-by-screening medium, Arabidopsis genomic DNA was extracted using the CTAB (Cetyltrimethylammonium bromide) method and tested using PCR. T3 plants from at least three independent lines were used for analysis.

### Determination of anthocyanin substances in *Arabidopsis thaliana* overexpressing *HvANS*

The experimental samples were sent to Novogene (Beijing, China) for analysis. The experimental samples were detected using multiple reaction monitoring (MRM) modes, and the compounds were quantified according to Q3 (daughter ion) and qualitatively analyzed by Q1 (parent ion), Q3 (daughter ion), retention time (RT), de-clustering voltage (DP), and collision energy (CE). SCIEX OSV1.4 software was used to open the downstream mass spectrometry files for peak integration and correction, and the peaks were screened according to a set minimum peak height of 500, signal-to-noise ratio of 5, number of smoothing points of 1, and so on. The area of the sub-ion peaks integrated in each chromatographic retention time represented the relative content of the corresponding substance. Finally, the integrated peak areas of all peaks were exported to obtain the qualitative and quantitative results of the metabolites.

The identified metabolites were annotated using the KEGG (https://www.genome.jp/kegg/pathway.html), HMDB (https://hmdb.ca/metabolites), and LIPIDMaps databases (http://www.lipidmaps.org/).

SPSS 27 software was utilized to determine the significance of anthocyanin substance content.

### Yeast one-hybrid (Y1H) assay

The full-length CDS of *HvnANT1* was recombined into the pGADT7 prey vector, and the promoter of *HvANS* was recombined into the pAbAi decoy vector. The recombinant plasmid was transformed into yeast (Y1H) cells. pANS-AbAi was grown on SD-Ura solid medium with different Aureobasidin A (AbA, TaKaRa, Ohtsu, Japan) concentrations (0, 100, 150, 200, 600, 800, and 1000 ng/mL) for 3 days at 30 °C, and its lowest inhibitory concentration was recorded. The p53-AbAi receptor state single-transformed pGADT7-Rec53 plasmid was used as the positive control. The pANS-AbAi receptor state was transfected with pGADT7 and pGADT7-ANT1 plasmids alone, and the former was used as a negative control and the latter as the experimental group. After transformation, the bacterial solution was spread on SD-Leu plates without AbA and SD-Leu plates containing the lowest inhibitory concentration of AbA and incubated at 30 °C for 4–5 days for observation.

### Yeast two-hybrid (Y2H) assay

Oebiotech (Shanghai, China) constructed a yeast library by cloning cDNA libraries of mRNAs from the PC1, PC2, and PC3 seed coats of Nierumuzha into pGADT7. The cDNA of HvANS was inserted into pGBKT7. Yeast library screening was performed using pGBKT7-HvANS according to the manufacturer’s manual (Clontech, Palo Alto, CA, USA). The full-length CDSs of *HvANS* and other genes were cloned into the pGBKT7 and pGADT7 vectors, respectively. The bait and prey constructs were co-transformed into yeast strain AH109 using the lithium acetate method. The transformants were cultured on SD/-Trp/-Leu/-His/-Ade/X-α-Gal/AbA plates in an incubator at 30 °C for 3–5 days. Positive clones were expected to grow and be blue.

### Electronic supplementary material

Below is the link to the electronic supplementary material.

Supplementary Material 1

Supplementary Material 2

Supplementary Material 3

Supplementary Material 4

## Data Availability

No datasets were generated or analysed during the current study.

## Abbreviations

PAs: Anthocyanins and proanthocyanidins
ANS: Anthocyanidin synthase
LDOX: Leucoanthocyanidin dioxygenase
TFs: Transcription factors
qRT-PCR: Quantitative reverse transcription polymerase chain reaction
PCR: Polymerase chain reaction
CDS: Coding sequences
Y1H: Yeast one-hybrid
Y2H: Yeast two-hybrid
