# Supplementary material for: Unveiling the mysteries of HvANS: a study on anthocyanin biosynthesis in qingke (hordeum vulgare L. var. Nudum hook. f.) seeds
Source: BMC Plant Biol. 2024 Jul 6;24:637. doi: 10.1186/s12870-024-05364-2 (PMC11227189; doi:10.1186/s12870-024-05364-2)
Supplement: Supplementary file 1 — Supplementary Material 1 [file 12870_2024_5364_MOESM1_ESM.docx]

**Table S1** Structure of cis-acting elements in the promoter region of *HvANS* gene

| Site name | Sequence | Number | Function |
| --- | --- | --- | --- |
| A-box | CCGTCC | 1 | cis-acting regulatory element |
| ABRE | ACGTG/AACCCGG/CACGTG | 5 | cis-acting element involved in the abscisic acid responsiveness |
| ABRE3a | TACGTG | 1 | Cis-acting elements associated with growth and development, hormonal signalling and response to stress |
| ABRE4 | CACGTA | 1 | Cis-acting elements associated with growth and development, hormonal signalling and response to stress |
| AE-box | AGAAACAA | 2 | part of a module for light response |
| ARE | AAACCA | 2 | a cis-acting regulatory element essential for the anaerobic induction |
| AT-rich element | ATAGAAATCAA | 1 | binding site of AT-rich DNA binding protein (ATBP-1) |
| AT~TATA-box | TATATA | 3 |  |
| Box 4 | ATTAAT | 1 | part of a conserved DNA module involved in light responsiveness |
| CAAT-box | CAAT/ CCAAT/ CAAAT | 22 | common cis-acting element in promoter and enhancer regions |
| CAT-box | GCCACT | 1 | cis-acting regulatory element related to meristem expression |
| CCAAT-box | CAACGG | 1 | MYBHv1 binding site |
| CGTCA-motif | CGTCA | 3 | cis-acting regulatory element involved in the MeJA-responsiveness |
| G-Box | CACGTG | 1 | cis-acting regulatory element involved in light responsiveness |
| G-box | CACGTC/ TACGTG/ CACGAC | 5 | cis-acting regulatory element involved in light responsiveness |
| GATA-motif | AAGGATAAGG | 1 | part of a light responsive element |
| GCN4_motif | TGAGTCA | 1 | cis-regulatory element involved in endosperm expression |
| LTR | CCGAAA | 4 | cis-acting element involved in low-temperature responsiveness |
| MYB | CAACCA/ CAACAG | 7 | Involvement in the regulation of secondary metabolic pathways in plant phenolpropanoids |
| MYB recognition site | CCGTTG | 1 | MYB recognition site |
| MYB-like sequence | TAACCA | 2 | Involvement in the regulation of secondary metabolic pathways in plant phenolpropanoids |
| Myb-binding site | CAACAG | 1 | MYB recognition site |
| TATA-box | TATATTTATATTT/ ATATAA/ TATA/ TAAAGATT/ TATATAAATC/ ATATAT/ TACAAAA/ TATATA/ TATAA/ TATAAAT/ TATAAA/ CCTATAAAAA | 33 | core promoter element around -30 of transcription start |
| TCA-element | CCATCTTTTT | 1 | cis-acting element involved in salicylic acid responsiveness |
| TCCC-motif | TCTCCCT | 2 | part of a light responsive element |
| TGACG-motif | TGACG | 3 | cis-acting regulatory element involved in the MeJA-responsiveness |
| chs-Unit 1 m1 | ACCTAACCCGG | 1 | cis-acting regulatory element involved in the MeJA-responsiveness |
| motif I | GGTACGTGGCG | 1 | cis-acting regulatory element root specific |
