# Supplementary figures and images for: Unveiling the mysteries of HvANS: a study on anthocyanin biosynthesis in qingke (hordeum vulgare L. var. Nudum hook. f.) seeds
Source: BMC Plant Biol. 2024 Jul 6;24:637. doi: 10.1186/s12870-024-05364-2 (PMC11227189; doi:10.1186/s12870-024-05364-2)

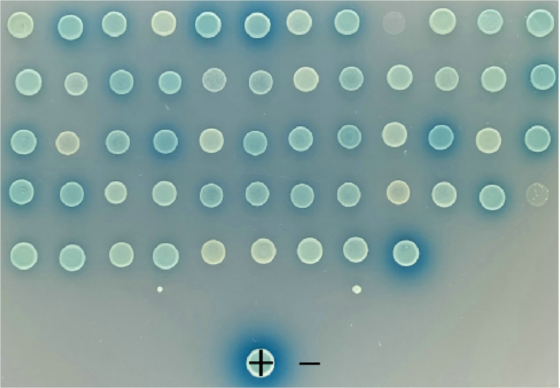


Fig.S2. Results of the nuclear system two-hybrid re-screening library.

Supplement: Supplementary file 2 — Supplementary Material 2 [file 12870_2024_5364_MOESM2_ESM.docx]

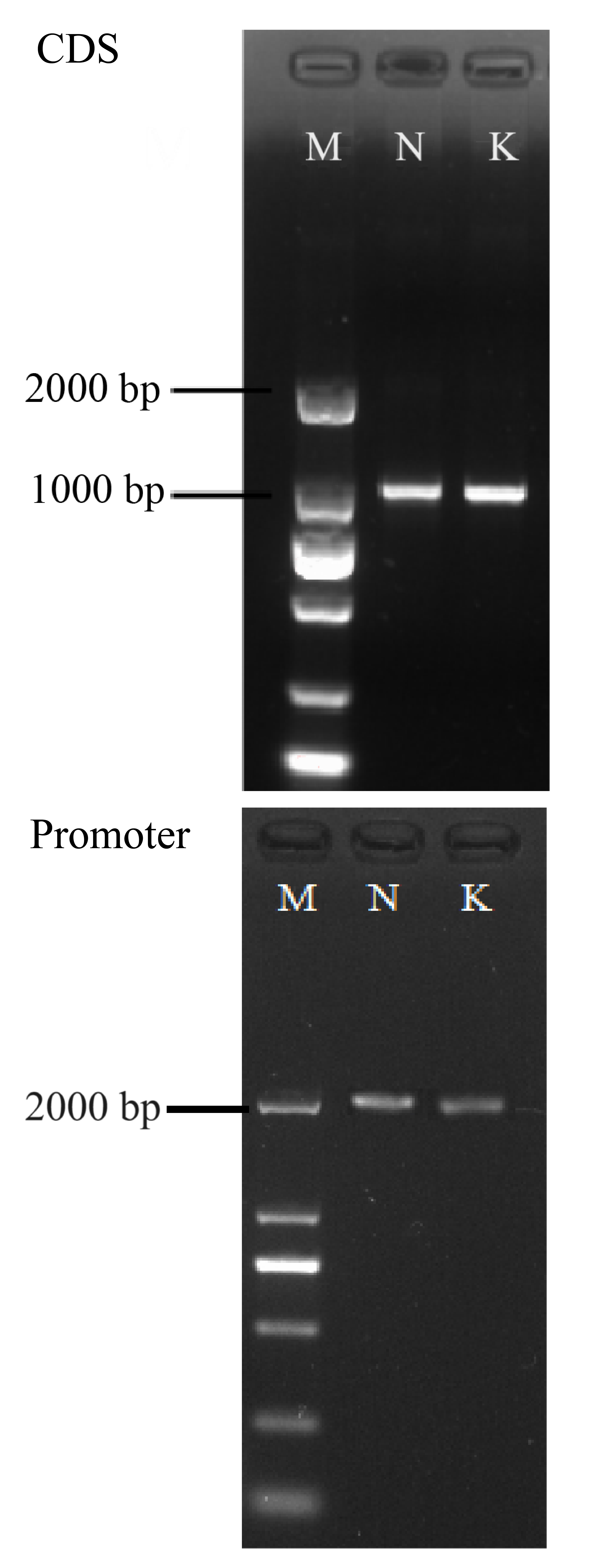

Supplement: Supplementary file 3 — Supplementary Material 3 [file 12870_2024_5364_MOESM3_ESM.png]
