# Supplementary material for: Unveiling the mysteries of HvANS: a study on anthocyanin biosynthesis in qingke (hordeum vulgare L. var. Nudum hook. f.) seeds
Source: BMC Plant Biol. 2024 Jul 6;24:637. doi: 10.1186/s12870-024-05364-2 (PMC11227189; doi:10.1186/s12870-024-05364-2)
Supplement: Supplementary file 4 — Supplementary Material 4 [file 12870_2024_5364_MOESM4_ESM.docx]

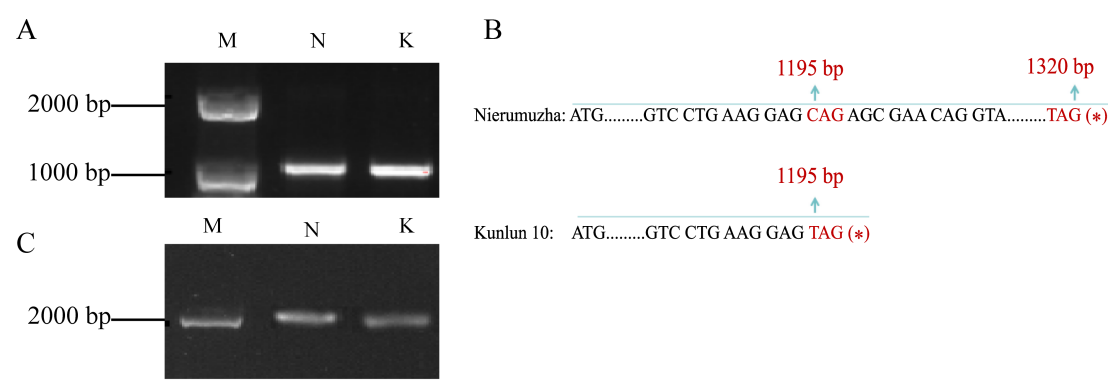


Fig. S1 **Amplification products of the coding sequence (CDS) and the promoters of the *HvANS* gene and CDS sequences.** (**A**) PCR amplified product of the *HvANS* gene. (**B**) CDS of the *HvANS* gene in two species. (**C**) Promoter amplification products of the *HvANS* gene in two species. N: Nierumuzha; K: Kunlun 10; M: Marker; *: stop codon.


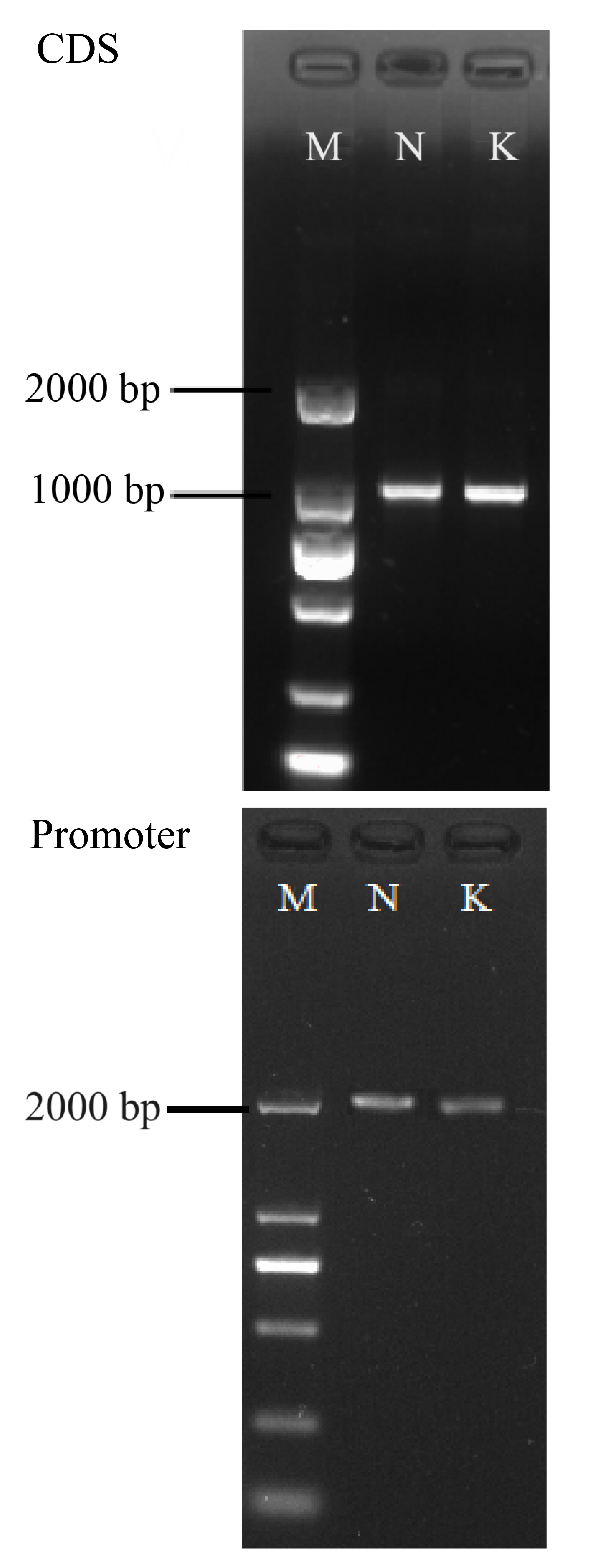


the full uncropped Gels and Blots image
